# Bardoxolone methyl improves survival and reduces clinical measures of kidney injury in tumor-bearing mice treated with cisplatin

**DOI:** 10.1186/s41120-025-00107-5

**Published:** 2025-03-03

**Authors:** Lauren E. Thompson, Stacey M. Tuey, Paola Garcia Gonzalez, Carly S. Chesterman, Courtney D. McGinnis, M. Scott Lucia, Lauren M. Aleksunes, Charles L. Edelstein, Melanie S. Joy

**Affiliations:** 1 Department of Pharmaceutical Sciences, Skaggs School of Pharmacy and Pharmaceutical Sciences, University of Colorado Anschutz Medical Campus, Aurora, CO, USA.; 2 Department of Pathology, University of Colorado Anschutz Medical Campus, Aurora, CO, USA.; 3 Division of Renal Diseases and Hypertension, University of Colorado Anschutz Medical Campus, Aurora, CO, USA.; 4 University of Colorado Cancer Center, University of Colorado Anschutz Medical Campus, Aurora, CO, USA.; 5 Environmental and Occupational Health Sciences Institute, Rutgers University, Piscataway, NJ, USA.; 6 Department of Pharmacology and Toxicology, Ernest Mario School of Pharmacy, Rutgers University, Piscataway, NJ, USA.

**Keywords:** Cisplatin, Nephrotoxicity, Bardoxolone Methyl, Acute Kidney Injury, Nrf2

## Abstract

**Objective:**

Acute kidney injury (AKI) occurs in approximately one-third of patients treated with cisplatin and there is an outstanding need for mitigation strategies to decrease the frequency and severity of cisplatin-induced AKI. This study evaluated bardoxolone methyl (BARD) as a nephroprotectant in a multidose, tumor-bearing mouse model of cisplatin-induced AKI. BARD is an attractive therapeutic intervention due to its ability to protect against cisplatin-induced nephrotoxicity by activating Nrf2 and previous reports suggesting anti-tumorigenic effects.

**Methods:**

In this study, CMT167 tumor-bearing mice were treated with four weekly doses of cisplatin with or without BARD and evaluated for survival, tumor growth, and clinical and histological measures of AKI. Kidney injury and/or function were evaluated by quantification of urinary kidney injury molecule-1 (KIM-1) and serum creatinine (SCr) levels as well as histopathology.

**Results:**

Compared to mice receiving cisplatin alone, co-treatment with BARD significantly enhanced survival (*p* = 0.01). Moreover, BARD prevented elevation of urinary KIM-1 concentrations as early as one week after cisplatin treatment (*p* < 0.01) – a response that was observed throughout the 4-week study period. Cisplatin increased SCr concentrations by four weeks, which was prevented by BARD co-administration (*p* < 0.01). Cisplatin treatment significantly decreased tumor burden compared to vehicle-treated mice (*p* < 0.05 after two cisplatin doses) – a response that was not altered by BARD co-treatment.

**Conclusions:**

Overall, the results of this study demonstrate that BARD has the potential to improve survival and reduce clinical measures of kidney injury in tumor-bearing mice treated with cisplatin, suggesting it could be used as a nephroprotectant to mitigate cisplatin-induced AKI.

## Introduction

Cisplatin, a platinum-based chemotherapeutic first approved by the U.S. Food and Drug Administration (FDA) in 1978, is used to treat many types of solid tumors. However, despite almost 50 years of use and continued research, over 30% of cisplatin-treated patients develop acute kidney injury (AKI) as a major side effect of treatment (Akcay et al. 2010; George et al. 2018; Einhorn 2002). Mouse models to study cisplatin kidney injury have largely relied on a single high dose (20–40 mg/kg) that resulted in severe kidney injury and death within 3–7 days (Oh et al. 2014). Beginning in 2016, multidose cisplatin mouse models were developed to more closely recapitulate the repeated dosing used in humans across multiple cycles (Ravichandran et al. 2016; Ravichandran et al. 2018; Brown et al. 2020; Sharp et al. 2018; Katagiri et al. 2016; Ozkok and Edelstein 2014; Holditch et al. 2019; Torres et al. 2016; Christian 2016). Sub-chronic mouse models utilize 5–15 mg/kg cisplatin administered once weekly for 2–4 weeks (Ravichandran et al. 2016; Ravichandran et al. 2018; Brown et al. 2020; Sharp et al. 2018; Sadhukhan et al. 2018; Solanki et al. 2015). However, neither the traditional single dose nor the multidose cisplatin regimens typically employ mice that have tumors, limiting the full translation to oncology patients. Tumor-bearing models have the advantage of allowing for analysis of responses to treatment and kidney injury in the presence of tumors. Cancer cells including CMT167, a murine pulmonary adenocarcinoma cell line, have been used for tumor engraftment in multidose cisplatin-induced AKI mouse models (Ravichandran et al. 2016; Ravichandran 2018; Brown et al. 2020). Repeated administration of cisplatin to tumor-bearing mice most closely recapitulates cisplatin-induced AKI in cancer patients and represents a preclinical model that can be used to test potential mitigation strategies.

**Fig. 1:**
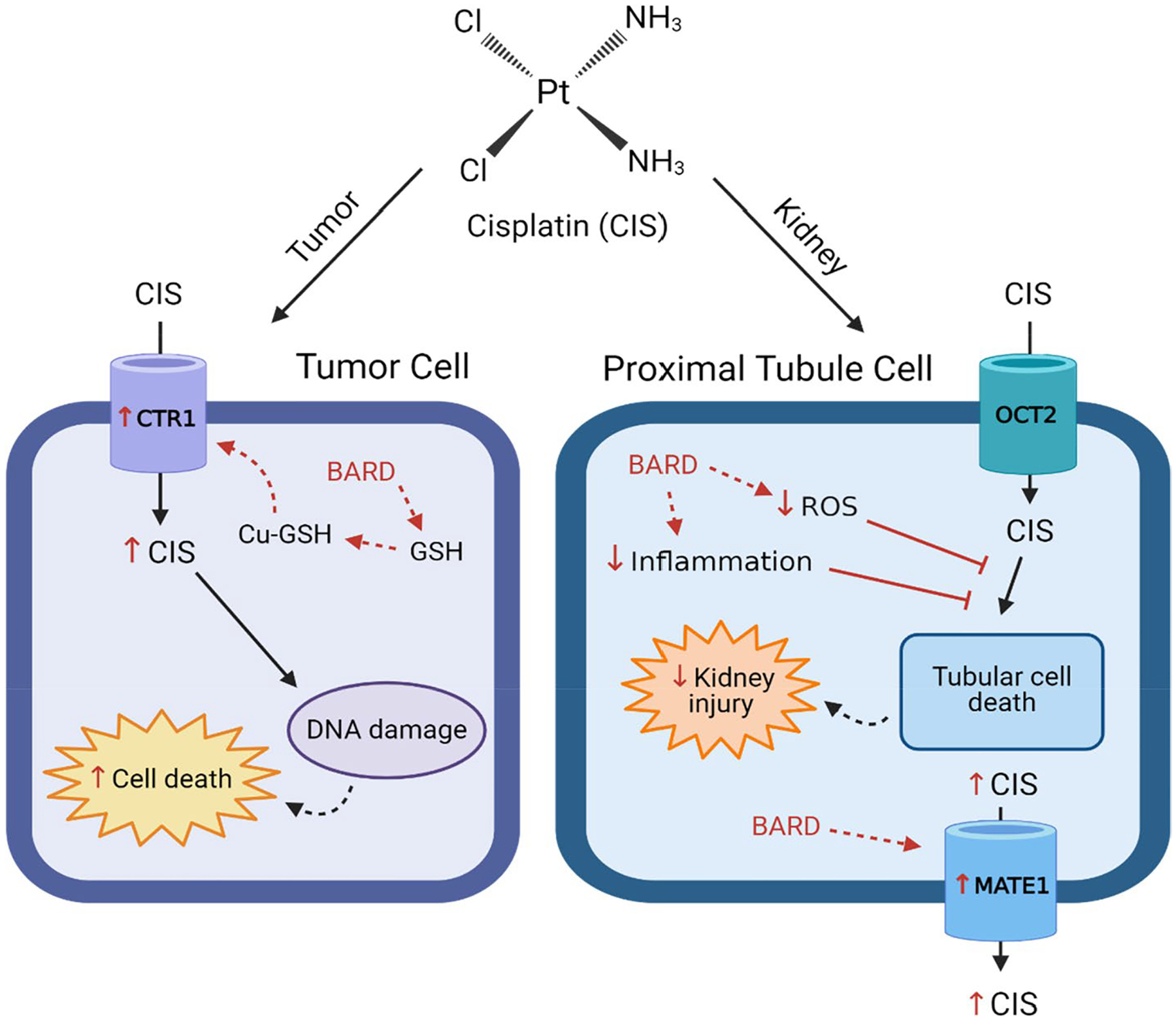
Effects of bardoxolone methyl (BARD) (red indicators) in tumor cells and proximal tubule cells. BARD: bardoxolone methyl; CIS: cisplatin; CTR1: high affinity copper uptake protein 1; Cu: copper; GSH: glutathione; MATE1: multidrug and toxin extrusion protein 1; OCT2: organic cation transporter 2; ROS: radical oxygen species. Figure was made using BioRender.com

Over the years, experimental mitigation strategies for cisplatin-induced kidney injury have employed a multitude of compounds with varying levels of success, but few have been attempted in tumor-bearing mice receiving cisplatin across multiple cycles (Ravichandran et al. 2016; Ravichandran et al. 2018; Brown et al. 2020). Regarding mechanisms for kidney injury, oxidative stress and inflammation are particularly attractive mitigation pathways relevant for cisplatin-induced kidney injury (Ozkok and Edelstein 2014; McSweeney 2021). Bardoxolone methyl (BARD), or CDDO-Me, is a synthetic triterpenoid that is derived from oleanolic acid and serves as a potent nuclear factor erythroid 2-related factor 2 (Nrf2) activator that was shown in vitro to have protective effects against cisplatin-induced AKI by mitigating oxidative stress and inflammation (Fig. 1) (Aleksunes et al. 2010; Hasegawa 2017; Camer et al. 2016; Ruiz et al. 2013; Pergola et al. 2011a; Atilano-Roque et al. 2016a; Atilano-Roque et al. 2016b; Yoh et al. 2001; Kurosaki et al. 2022; Mapuskar et al. 2023). Also, *Nfe2l2*-null mice (*Nfe2l2* encodes Nrf2) exhibit enhanced nephrotoxicity following a single dose of cisplatin when compared to wildtype mice, suggesting that Nrf2 serves as a defense mechanism against cisplatin-induced AKI (Aleksunes et al. 2010). Nrf2 is normally bound by Kelch-like ECH-associated protein 1 (Keap1), which targets Nrf2 for continual proteasomal degradation. This shuttling to the proteasome limits the extent of Nrf2 translocation and binding to antioxidant response elements (ARE) in the promoter regions of target genes (Mapuskar et al. 2023; Itoh et al. 1997; Kohandel et al. 2021; Farkhondeh et al. 2020). Enhanced reactive oxygen species (ROS) can cause changes in the structure of Keap1, resulting in Keap1 losing the ability to bind Nrf2 (Mirzaei et al. 2021). BARD, a Nrf2 activator, can also pharmacologically facilitate the release of Nrf2 from Keap1 sequestration. Then, Nrf2 translocates to the nucleus and following heterodimerization with small Maf (musculoaponeurotic fibrosarcoma) proteins binds to ARE-containing genes, and recruits RNA polymerase and transcriptional machinery to up-regulate the expression of antioxidant and cytoprotective genes, such as glutamate-cysteine ligase catalytic subunit (*GCLC*) (Mapuskar et al. 2023; Mirzaei et al. 2021; Ammal Kaidery et al. 2019; Itoh et al. 2004). The activation of Nrf2 additionally suppresses nuclear factor kappa-light-chain-enhancer of activated B cells (NF-κB) activity, reducing cytokine production (Wardyn et al. 2015). An in vitro study in primary human proximal tubule epithelial cells showed that BARD (100 nM), administered either pre- or post-cisplatin exposure, enhanced cellular survival and expression of the cytoprotective genes, NAD(P)H quinone oxidoreductase 1 (*NQO1*) and *GCLC* (Atilano-Roque et al. 2016a). In addition to reduction of ROS and inflammation, BARD may exhibit favorable effects on tumors by inhibiting proliferation and stimulating differentiation and apoptosis in cancer cells (Fig.1) (Gao et al. 2015; Liby et al. 2010; Shishodia et al. 2006; Konopleva et al. 2005; Konopleva et al. 2002).

Preferred characteristics for a kidney injury prevention strategy for concomitant administration with cisplatin would be demonstration of reduced kidney injury as evidenced by biomarkers and/or functional assessments, and lack of interference with chemotherapy efficacy on tumor size reduction. The current study sought to explore the potential kidney and tumor benefits of BARD in a pilot study conducted over 4-weeks and employed a cisplatin mouse model with engrafted tumors. The overall goal was to establish the potential of BARD as a mitigation strategy for cisplatin-induced AKI.

## Methods

### Reagents

Cisplatin was purchased from Sigma-Aldrich (St. Louis, MO) and dissolved in saline (G-Biosciences, St. Louis, MO). BARD was obtained from MedChem Express (Monmouth Junction, NJ) and dissolved in 34.5% PEG-300 (Sigma-Aldrich, St. Louis, MO), 30% DMSO (Sigma-Aldrich, St. Louis, MO), 30% saline, 5% propylene glycol (Fisher Scientific, Hampton, NH), and 0.5% Tween-80 (Fisher Scientific, Hampton, NH). Dulbecco’s Modification of Eagle’s Medium (DMEM) with 1 g/L glucose, L-glutamine, and sodium pyruvate was purchased from Mediatech, Inc. (Manassas, VA). Fetal bovine serum and HEPES buffer solution were purchased from Fisher Scientific (Hampton, NH). MEM non-essential amino acids (100X), penicillin–streptomycin, and 0.25% trypsin–EDTA were purchased from Gibco (Waltham, MA).

### Cell line and culture conditions

CMT167 murine lung carcinoma cells that harbor an activating Kras^G12V^mutation and are derived from C57BL/6J mice were used to form tumors in the mouse model, as described below (Li et al. 2018). CMT167 cells were generously provided by Dr. Charles Edelstein and Dr. Raphael Nemenoff at the University of Colorado Anschutz Medical Campus. CMT167 cells were cultured and maintained in DMEM supplemented with 10% fetal bovine serum, 1% penicillin–streptomycin, 1% HEPES buffer, and 1% MEM non-essential amino acids in a cell incubator at an atmosphere of 5% CO_2_ and 95% relative humidity at 37 °C for all experimental procedures unless stated otherwise. All cells underwent two passages before use.

### Tumor-bearing cisplatin-AKI mouse model

A previously published tumor-bearing cisplatin mouse model was used for the experiments (Ravichandran et al. 2016; Ravichandran et al. 2018; Brown et al. 2020). For all studies, male 8–10 week old C57BL/6J mice (Jackson Laboratories, Bar Harbor, ME) weighing 20–30 g were used. Experiments were conducted in adherence to the NIH Guide for the Care and Use of Laboratory Animals. The animal protocol was approved by the Institutional Animal Care and Use Committee of the University of Colorado Anschutz Medical Campus. Mice were maintained on a standard chow diet and water was freely available. Mice were housed 4 per cage under a 12-h light and dark schedule for at least two weeks prior to cisplatin administration. Prior to study initiation, the mouse model (Ravichandran et al. 2016; Ravichandran et al. 2018; Brown et al. 2020) was authenticated for the number of CMT167 cancer cells to inject to form tumors, the time needed for tumor growth, and the cisplatin dosing to result in kidney injury (data not shown).

**Fig. 2:**
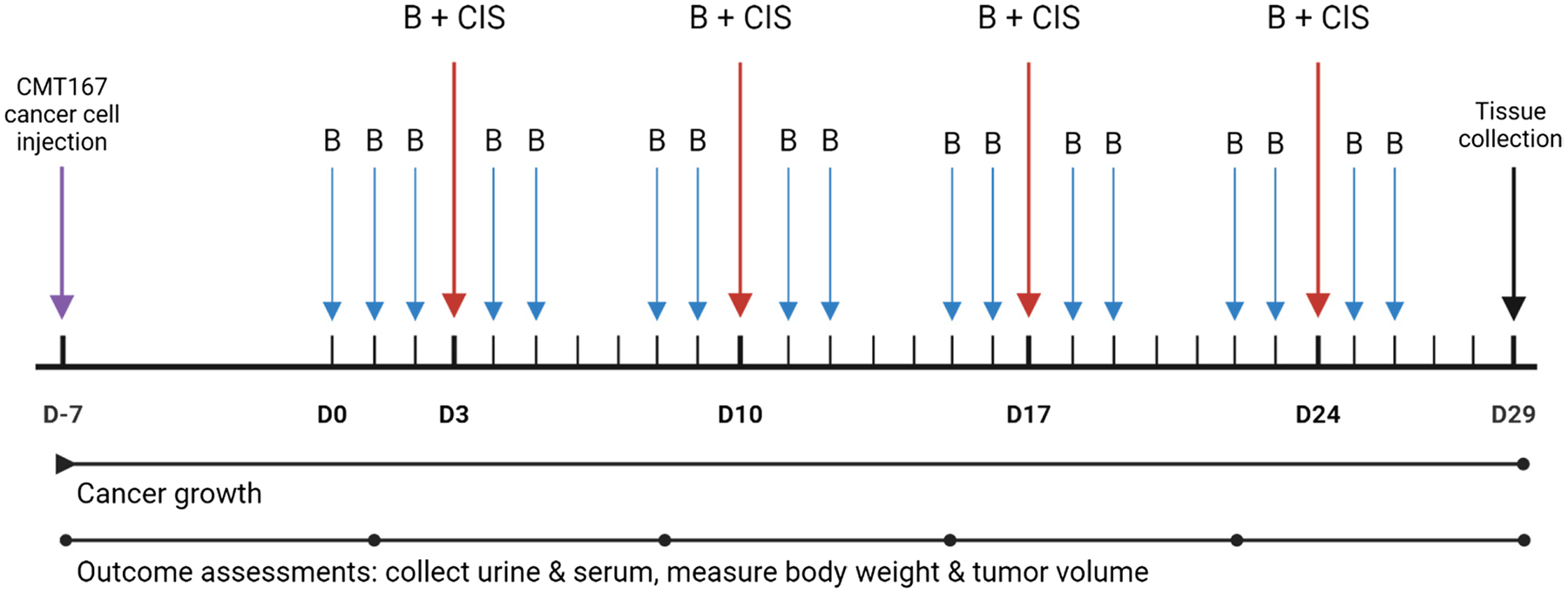
Cisplatin-induced acute kidney injury (AKI) mouse model timeline. Timeline of cisplatin-induced AKI mouse model assessing impact of BARD co-treatment. Each blue arrow indicates a dose of BARD (10 mg/kg) or BARD vehicle. Each red arrow indicates a dose of cisplatin (10–15 mg/kg) or cisplatin vehicle along with a dose of BARD (10 mg/kg) or BARD vehicle. Outcome assessments were performed weekly and included the collection of urine and serum and the measurement of body weight and tumor volume. D: day; B: BARD, CIS: cisplatin. Figure was made using BioRender.com

In the mouse model, CMT167 cells were injected s.c. (50,000 cells in DMEM media) with a 26-g needle into the right flank. Tumors were allowed to engraft for 7 days before treatment was initiated (Fig. 2). After tumor engraftment, BARD 10 mg/kg or vehicle (34.5% PEG-300, 30% DMSO, 30% saline, 5% propylene glycol, and 0.5% Tween-80) was injected i.p. five times per week and cisplatin 10–15 mg/kg or vehicle (saline) was injected i.p. once weekly for four weeks (Fig. 2). The cisplatin dose was decreased from 15 mg/kg to 10 mg/kg following the first two doses of cisplatin due to a high level of observed toxicity and reduced survival. Mice were randomized into four treatment groups (Veh/Veh, BARD/Veh, CIS/Veh, BARD/CIS) with 8–16 animals per group, with higher numbers in CIS groups to ensure adequate numbers given this toxic chemotherapeutic (Table S1). Body weight and tumor size at the start of treatment were not significantly different between the treatment groups (Table S1). Mice were administered saline subcutaneously on the days before and after cisplatin treatment to prevent dehydration.

Urine and blood (by submandibular bleed) were collected at the time of cancer cell injection, and weeks 1, 2, 3, and 4 of treatment. At baseline and weeks 1, 2, 3, and 4 of treatment, tumor size was quantitated using digital calipers and body weight was measured. After four weeks of treatment, the mice were euthanized by isoflurane anesthetization and exsanguination, urine and blood were collected, and tumors and kidneys were excised and weighed. Each kidney was weighed separately.

### Sample assays

Urinary kidney injury molecule-1 (KIM-1) was measured using a mouse KIM-1 Enzyme-Linked Immunosorbent Assay (ELISA) Kit (Cat. #80652, Crystal Chem, Elk Grove Village, IL). Serum creatinine (SCr) was measured using a QuantiChrom Creatinine Assay Kit (Cat. #DICT-500, BioAssay Systems, Hayward, CA).

Mouse kidneys and tumors collected at the end of the study were homogenized in PBS (Gibco, Waltham, MA). Glutamate-cysteine ligase catalytic subunit (GCLC) protein levels were measured in the homogenized kidney and tumor samples (GCLC ELISA Kit (Mouse), Cat. #OKEH06983, Aviva Systems Biology, San Diego, CA).

### Statistical analysis

Survival curves for each group were compared using a log-rank (Mantel-Cox) test using GraphPad Prism (v10.0.2, San Diego, CA). Analyses for differences between treatment groups for tumor sizes, kidney weights, urinary KIM-1, SCr, and GCLC protein levels were assessed by two-way analysis of variance (ANOVA) with Tukey–Kramer post-hoc test using GraphPad Prism. Data are presented as mean ± standard deviation (SD). A *p* value of < 0.05 was considered statistically significant.

## Results

### BARD enhances survival of tumor-bearing mice treated with cisplatin

**Fig. 3:**
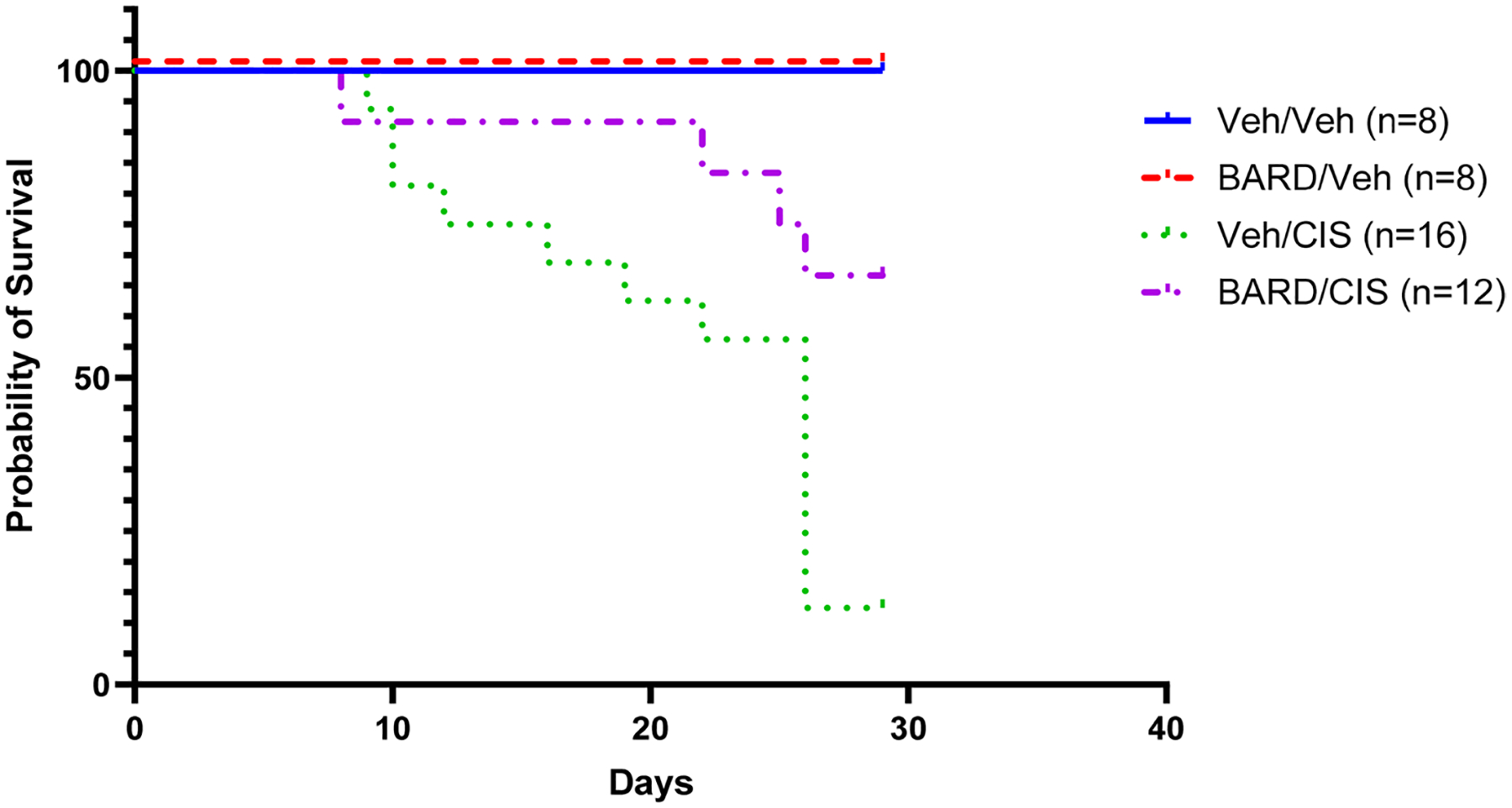
Bardoxolone methyl (BARD) prevents survival decline in cisplatin-treated mice. Survival curve for each treatment group of mice (*n* = 8–16/group). The Veh/Veh and BARD/Veh groups had 100% survival from the first day of BARD dosing (day 0) until study end (day 29). The Veh/CIS group experienced a significantly larger decrease in survival when compared to each of the other treatment groups (*p* < 0.05), including the BARD/CIS group (*p* = 0.01)

The Veh/Veh and BARD/Veh groups had 100% survival at the planned study end on day 29 (Fig. 3). The Veh/CIS group demonstrated a significantly larger decline in survival (13% survival at study end) when compared to each of the other treatment groups (*p* < 0.05), including the BARD/CIS group (67% survival at study end; *p* = 0.01; Fig. 3). The addition of BARD prevented premature death in cisplatin-treated mice with cancer.

### BARD protects against kidney injury in tumor-bearing mice treated with cisplatin

**Fig. 4:**
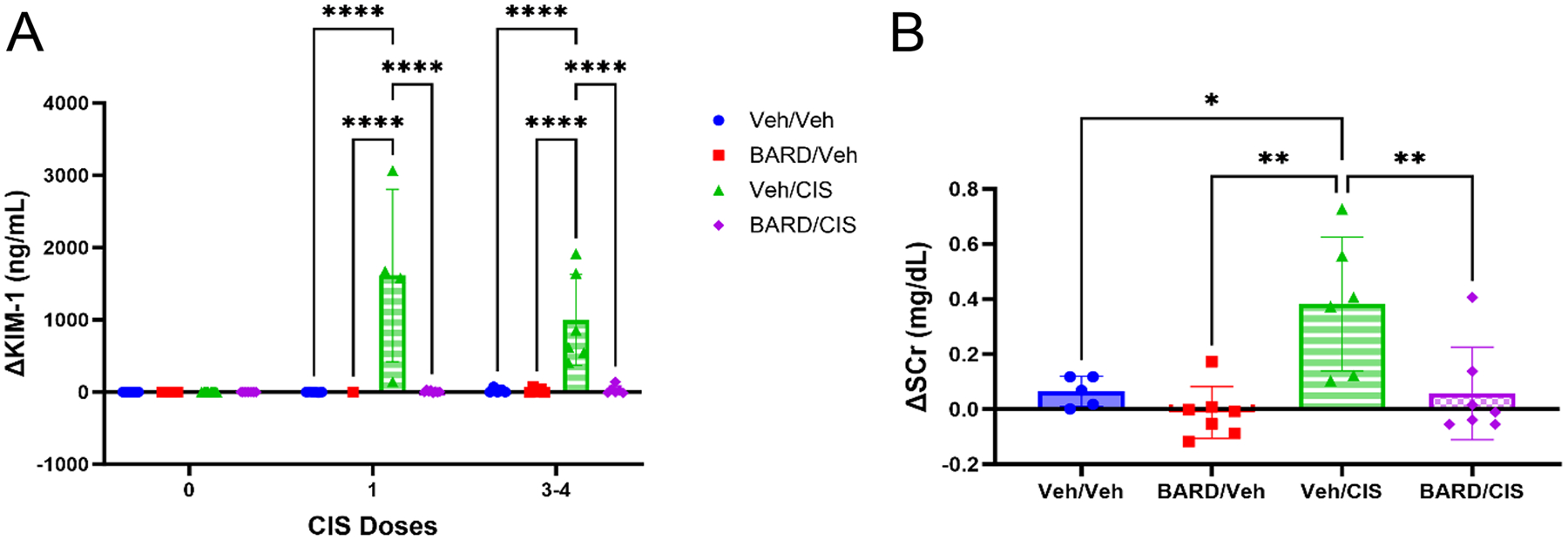
Bardoxolone methyl (BARD) co-treatment protects against cisplatin-induced acute kidney injury (AKI). **A** Average change in urinary kidney injury molecule-1 (KIM-1) levels from baseline for each treatment group (*n* = 1–14/group). **B** Average change in serum creatinine (SCr) levels from baseline for each treatment group (*n* = 5–7/group) after four doses of cisplatin. Data represented as mean ± standard deviation (SD); *p ≤ 0.05, **p ≤ 0.01, ****p ≤ 0.0001

Kidney damage was measured using urinary KIM-1 levels, histopathologic assessment of tubular injury and apoptosis, and kidney weights after four doses of cisplatin. Urinary KIM-1, a protein biomarker of proximal tubule injury, was measured in all available urine samples (Fig. 4A). Veh/Veh, BARD/Veh, and BARD/CIS groups did not exhibit any significant changes in urinary KIM-1 levels compared to baseline throughout the entirety of the study (*p* > 0.98). Conversely, the Veh/CIS group demonstrated a significant increase in urinary KIM-1 levels from baseline after just one dose of cisplatin (average 299-fold increase from baseline; *p* < 0.01) and these levels were significantly higher than all three other treatment groups (*p* < 0.01; Fig. 4A). After 3–4 doses of cisplatin, the Veh/CIS group maintained significantly higher urinary KIM-1 levels (average 72-fold increase from baseline) than the other three treatment groups (*p* < 0.01; Fig. 4A). Cisplatin-treated mice receiving BARD were protected from kidney injury as measured by KIM-1.

Mice in both the Veh/CIS and BARD/CIS groups demonstrated pathological tubular injury as assessed by histological changes (cell necrosis, loss of brush border, cast formation, tubule dilatation) in the outer stripe of the outer medulla (Fig. S1). All Veh/CIS treated mice exhibited tubular injury after just two doses of cisplatin as measured by urinary KIM-1 and had significantly higher pathological tubular injury scores than Veh/Veh and BARD/Veh mice (*p* < 0.05; Fig. S1A). Approximately 30% of BARD/CIS mice failed to exhibit tubular injury after receiving the four planned cisplatin doses. However, BARD co-treatment did not significantly alter tubular injury scores in cisplatin-treated mice overall (*p* = 0.53; Fig. S1A). Among the 70% of BARD/CIS mice that did exhibit tubular injury, there was not a statistical difference in tubular injury scores compared to the Veh/CIS mice (*p* > 0.99). Notably, kidneys from the mice that died prematurely were not available for histopathological analysis which included 5 in the Veh/CIS and 1 in the BARD/CIS groups.

The Veh/CIS group demonstrated non-significant increases in the number of apoptotic bodies in kidney tissue compared to the Veh/Veh (*p* = 0.25), BARD/Veh (*p* = 0.12), and BARD/CIS groups (*p* = 0.92) (Fig. S1B). Some cisplatin-treated mice, both with and without BARD co-treatment, exhibited periodic acid-Schiff (PAS)-positive nuclear inclusions within tubular cells which was not observed in vehicle-treated mice (Fig. S1E).

Mice treated with cisplatin had significantly smaller kidneys (124 ± 20 mg) as compared to non-cisplatin-treated mice (161 ± 24 mg; *p* < 0.0001; Fig. S2). BARD co-treatment did not alter kidney weights in cisplatin-treated mice (Veh/CIS: 119 ± 16 mg; BARD/CIS: 129 ± 23 mg; *p* = 0.50; Fig. S2). BARD co-treatment with cisplatin showed a protective effect on cisplatin kidney injury as assessed by urinary KIM-1 and histopathology.

### BARD mitigates increases in SCr in tumor-bearing mice treated with cisplatin

Kidney function was evaluated using the traditional clinical marker SCr. SCr was measured following four cisplatin doses. The Veh/CIS group had ~ twofold significantly higher SCr levels (0.752 ± 0.247 mg/dL) when compared to the Veh/Veh (0.414 ± 0.055 mg/dL), BARD/Veh (0.337 ± 0.094 mg/dL), and BARD/CIS (0.406 ± 0.169 mg/dL) treatment groups (*p* < 0.05; Fig. 4B).

### BARD may exhibit nephroprotection through Nrf2 activation and NF-κB inhibition

**Fig. 5:**
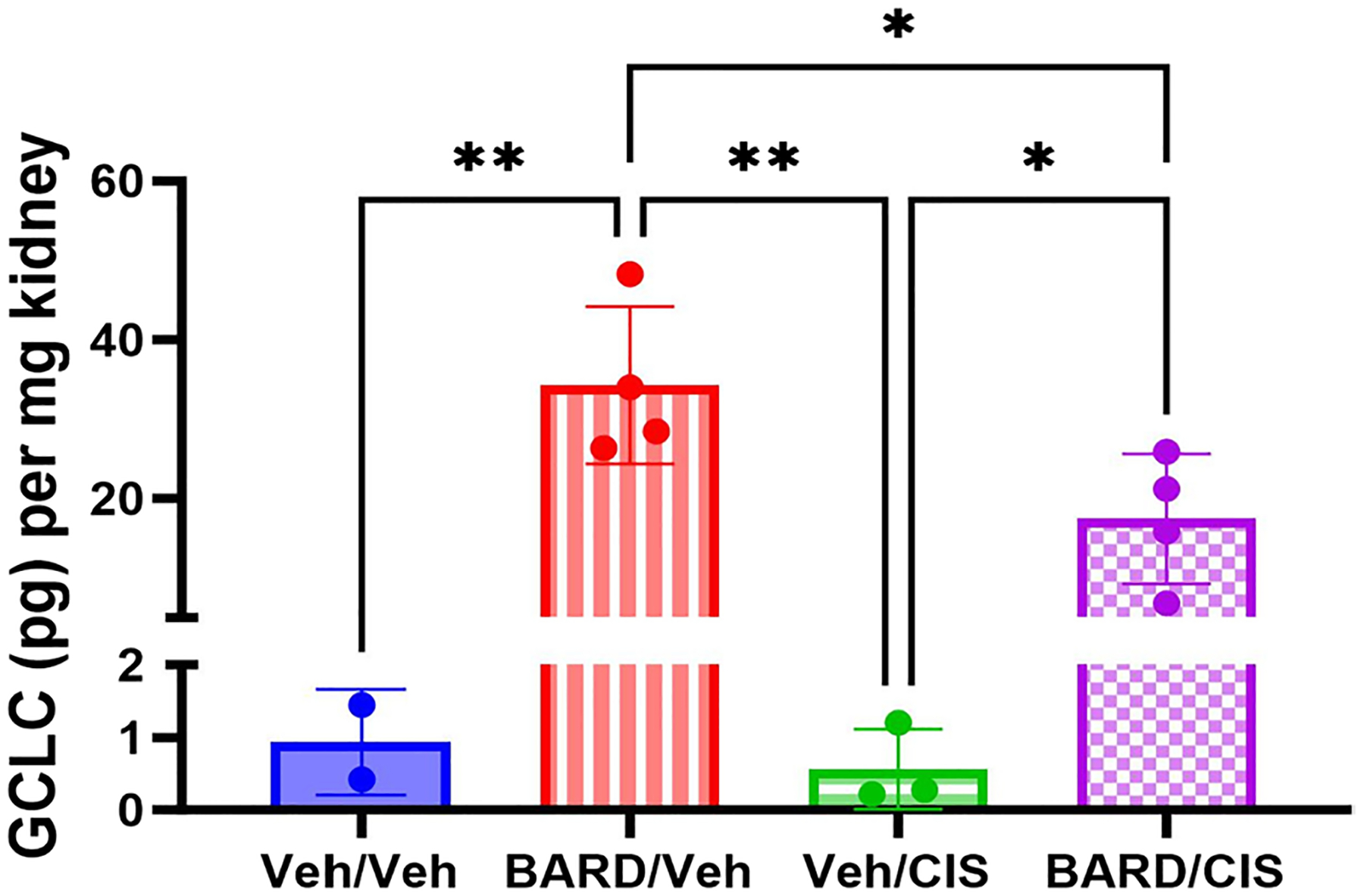
Bardoxolone methyl (BARD) increases GCLC protein levels in kidneys. Average glutamate cysteine ligase catalytic subunit (GCLC) protein levels in kidneys from each treatment group after four doses of cisplatin (*n* = 2–4/group). GCLC was quantified as pg GCLC per mg of kidney tissue. Data represented as mean ± standard deviation (SD); *p ≤ 0.05, **p ≤ 0.01

In order to confirm the presence of a kidney antioxidant state in the mouse model secondary to the mechanism of action of BARD, GCLC protein levels were measured in kidney samples collected at the study end after four doses of cisplatin. The BARD/Veh group exhibited increased GCLC in kidney tissue compared to the Veh/Veh (*p* < 0.01) and Veh/CIS (*p* < 0.01) groups (Fig. 5). The BARD/CIS group exhibited increased GCLC in kidney tissue compared to the Veh/CIS group (*p* < 0.05) (Fig. 5). The BARD/Veh group demonstrated a 2-fold increase in GCLC in kidney tissue compared to the BARD/CIS group (*p* < 0.05; Fig. 5). Low levels of GCLC protein (0.1–0.2 pg/mg kidney) were detected in tumor samples regardless of treatment group following four doses of cisplatin (data not shown).

Nrf2 activation was measured in both kidney and tumor tissues using a DNA-binding ELISA (Fig. S3A). Although there were no significant differences in Nrf2 activation in the kidneys, treatment with BARD resulted in non-significantly increased Nrf2 activation (*p* = 0.23; Fig. S3A). Cisplatin treatment resulted in a 2.2-fold increase in Nrf2 activation in tumor tissue compared to vehicle-treated mice (*p* < 0.01; Fig. S3A). The Veh/CIS and BARD/CIS groups displayed significantly increased Nrf2 activation in tumor tissue compared to the Veh/Veh group (*p* < 0.01; Fig. S3A).

NF-κB p50 and p65 activation were measured in both kidney and tumor tissues using a DNA-binding ELISA (Fig. S3B–C). Treatment with BARD resulted in a 1.3-fold increase in NF-κB p50 activation in the kidneys (*p* = 0.04; Fig. S3B). The BARD/Veh group exhibited a significant increase in NF-κB p50 activation in the kidneys compared to the Veh/Veh (*p* = 0.01) and BARD/CIS (*p* = 0.02) groups (Fig. S3B). No significant differences in NF-κB p50 activation in the tumors were observed (Fig. S3B). Treatment with cisplatin resulted in a 1.5-fold increase in NF-κB p65 activation in the kidneys (*p* < 0.01; Fig. S3C). The Veh/CIS group displayed a significant increase in NF-κB p65 activation in the kidneys compared to the Veh/Veh (*p* = 0.04) and BARD/Veh (*p* = 0.02) groups (Fig. S3C). The Veh/CIS group also displayed increased NF-κB p65 activation in the tumors compared to the Veh/Veh (*p* = 0.42), BARD/Veh (*p* = 0.26), and BARD/CIS (*p* = 0.23) groups (Fig. S3C). Treatment with BARD increased GCLC protein levels and NF-κB p50 activation in the kidneys, while cisplatin treatment increased tumor Nrf2 activation and NF-κB p65 activation in the kidneys.

### BARD co-treatment maintains anti-tumorigenic effects in tumor-bearing mice treated with cisplatin

**Fig. 6:**
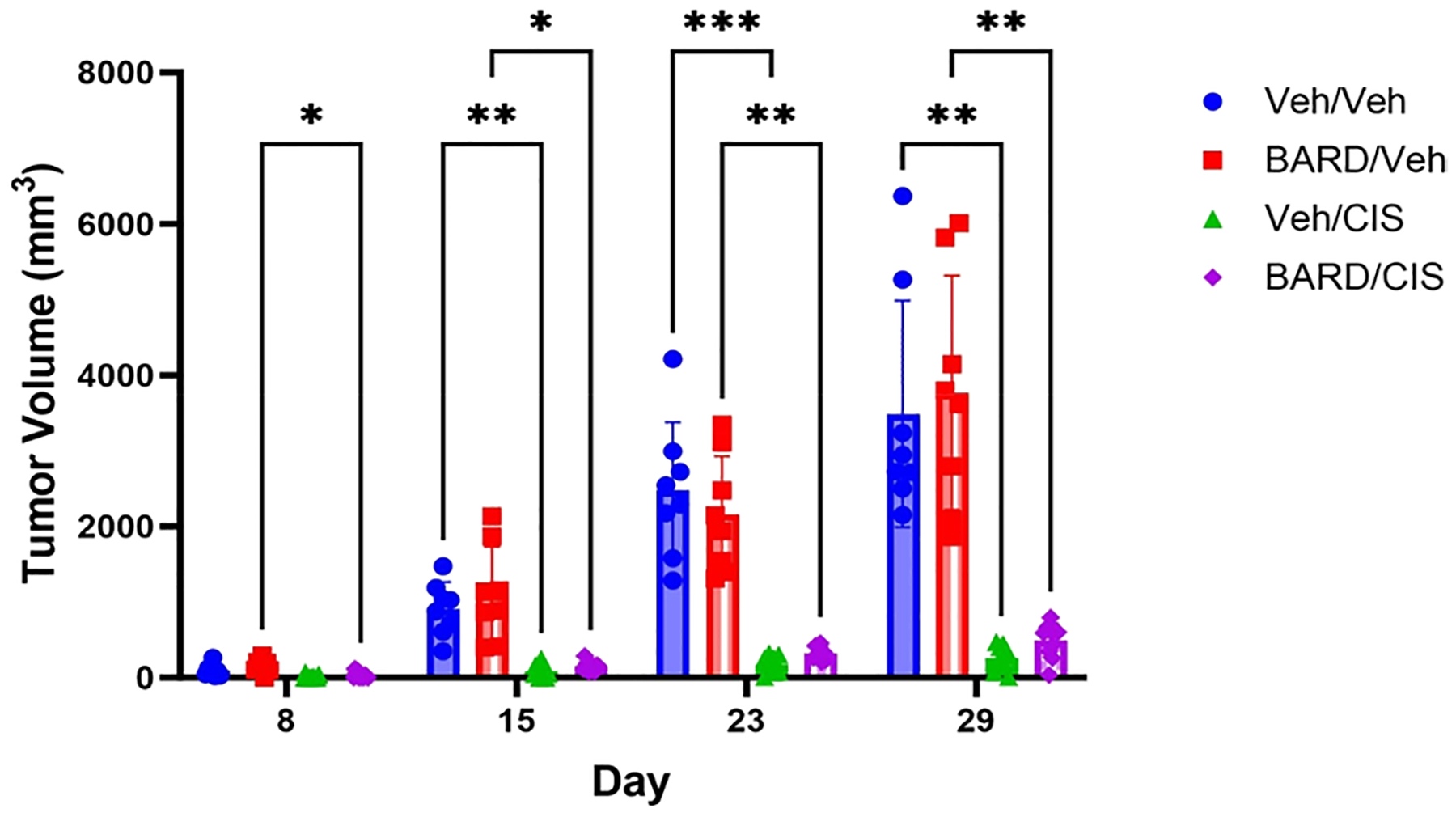
Bardoxolone methyl (BARD) does not alter cisplatin anti-tumor efficacy. Tumor volume for each of the treatment groups (*n* = 8–16/group). Data represented as mean ± standard deviation (SD); *p ≤ 0.05, **p ≤ 0.01, ***p ≤ 0.001

The Veh/CIS and BARD/CIS groups had significantly smaller tumors compared to the BARD/Veh group by day 8 (*p* < 0.05) and the Veh/Veh group by day 15 (*p* < 0.01; Fig. 6). There were no significant differences between the Veh/CIS and BARD/CIS groups (*p* > 0.05), indicating that co-treatment with BARD does not alter the anti-tumorigenic effects of cisplatin (Fig. 6). However, the BARD/CIS group did have a tumor volume ~ 2-fold greater at baseline than the Veh/CIS group (Table S1).

## Discussion

Approximately one-third of cisplatin-treated cancer patients develop AKI as a direct result of this treatment. Increasing evidence has shown that AKI can be a predictor of future clinical consequences, such as susceptibility to subsequent injury, chronic kidney disease, cardiovascular events, and mortality (Parr et al. 2016; Hoste et al. 2006). Mitigation strategies, such as nephroprotectant co-treatments, are needed to decrease the frequency and severity of cisplatin-induced acute nephrotoxicity to reduce the risk of subsequent chronic kidney disease. The current research sought to conduct a pilot study to investigate the effects of BARD on AKI secondary to cisplatin by studying a mouse model with engrafted tumors receiving four weekly cisplatin doses to closely recapitulate the scenario of clinical cancer patients receiving multiple cisplatin doses. Results from the study demonstrated that BARD significantly enhanced survival in cisplatin-treated mice, mitigated cisplatin-induced kidney injury, reduced loss of kidney function, and did not interfere with the anti-tumor effects of cisplatin. BARD enhanced expression of the GCLC protein in the kidneys, but not in the tumor tissue, suggesting that BARD displayed a kidney specific antioxidant mechanism to protect against cisplatin-induced AKI through an increase in this protein.

### Mitigation strategies for cisplatin-induced AKI

Previous mitigation strategies for cisplatin kidney injury have included other Nrf2 activators (i.e. CDDO-Im (Aleksunes et al. 2010), sulforaphane (Atilano-Roque et al. 2016b; Wen et al. 2015), oltipraz (Atilano-Roque et al. 2016b), oleanolic acid (Atilano-Roque et al. 2016b)), NF-κB inhibitors (i.e. JSH-23 (Ozkok et al. 2016)), TNF-α inhibitors (i.e. salicylate (Ramesh et al. 2004)), organic cation transporter 2 (OCT2) inhibitors (i.e. cimetidine (Katsuda et al. 2010)), MEK1/2 inhibitors (i.e. trametinib (Brown et al. 2020), U0126 (Brown et al. 2020)), cytoprotective/antioxidant agents (i.e. amifostine (Mercantepe et al 2018; Capizzi et al. 1999), carvedilol (Carvalho Rodrigues et al. 2012)), monoclonal antibodies (i.e. anti-CD4 T cells (Ravichandran et al. 2016)), magnesium (Solanki et al. 2015; Kumar et al. 2017), and gene knockout strategies (i.e.*Slc22a2*/OCT2 (Filipski et al. 2009),*Tnf-α*/TNF-α (Zhang et al. 2007), *Il-33*/IL-33 (Ravichandran et al. 2018), *Prx1*/Prx1 (Okada et al. 2013)), among others. The current study investigated BARD due to its potential ability to protect against cisplatin-induced nephrotoxicity by the dual mechanism of activating Nrf2 and inhibiting NF-κB (Aleksunes et al. 2010; Hasegawa et al. 2017; Camer et al. 2016; Ruiz et al. 2013; Pergola et al. 2011a; Atilano-Roque et al. 2016a, 2016b; Yoh et al. 2001; Kurosaki et al. 2022; Mapuskar et al. 2023; Wang et al. 2014), and previous reports suggesting it exhibits anti-tumorigenic effects (Gao et al. 2015; Liby et al. 2010; Shishodia et al. 2006; Konopleva et al. 2005, 2002). A similar Nrf2 activator, CDDO-Im, was previously tested in a non-tumor bearing, high single dose mouse model of cisplatin-induced AKI and resulted in increased Nrf2 signaling in the kidneys and protection from cisplatin nephrotoxicity as measured by changes in blood urea nitrogen and renal histopathology (Aleksunes et al. 2010).

BARD has been previously evaluated in clinical trials as a daily therapy to improve kidney function in patients with type 2 diabetes mellitus and chronic kidney disease (CKD). In these trials, daily treatment with BARD resulted in improvements in kidney function as measured by inulin clearance, estimated GFR (eGFR), and creatinine clearance (CrCl) (Pergola et al. 2011a; Chin et al. 2018; Pergola et al. 2011b; de Zeeuw et al. 2013). However, these clinical trials of BARD in diabetes and CKD patients were stopped early due to a signal of increased risk of heart failure (Chin et al. 2018). Post-hoc assessments reported that the primary reason for increased risk of heart failure was not direct cardiotoxicity by BARD, but rather due to fluid overload which was associated with two major risk factors: elevated baseline B-type natriuretic peptide and prior hospitalization for heart failure (Chin et al. 2018; de Zeeuw et al. 2013). Patients without these risk factors showed no increase in heart failure events or mortality when treated with BARD (Chin et al. 2018). BARD has never been tested in a clinical trial of cisplatin patients to determine whether acute therapy can mitigate kidney injury. As BARD use in these studies would be limited to acute administration at the time of chemotherapy, as opposed to chronic daily treatment required for patients with diabetes mellitus and CKD, this would naturally follow a risk-mitigation strategy for any of the previous fluid-related adverse effects. The limited use of BARD at the time of high kidney injury risk may decrease the risk of toxicity when compared to chronic usage and still provide a potential benefit in terms of reduction of kidney injury risk secondary to cisplatin. Further assessments are warranted to fully evaluate risks of fluid overload and heart failure in the preclinical model prior to the potential translation to cancer patients.

### Mouse models of cisplatin-induced AKI

There are two primary mouse models used to investigate cisplatin-induced AKI: the single high dose (20–40 mg/kg) model (Oh et al. 2014), and the multidose (5–15 mg/kg) model (Sharp et al 2018; Holditch et al. 2019). The single dose mouse model poorly replicates human clinical use of cisplatin as it uses a single lethal dose of cisplatin that results in mortality and nephrotoxicity 3–7 days following administration (Oh et al. 2014). A limitation to the multidose mouse models has been the lack of incorporation of animals with tumors. Translational models of cisplatin-induced kidney injury that utilize multiple doses of cisplatin administered to tumor-bearing mice have the propensity to better translate to the human clinical environment.

The mouse model in the current study used tumor-bearing mice receiving multiple cisplatin doses of 10–15 mg/kg for four weeks, which should best recapitulate cisplatin-induced AKI in cancer patients receiving cisplatin (Fig. 2). Minor changes (as described in the Methods) were made to the published mouse model (Ravichandran et al. 2016, 2018; Brown et al. 2020) in order to optimize the cisplatin dosing regimen and tumor growth protocol to measure kidney injury and survival in the male C57BL/6J mice used in this study.

### BARD co-treatment protects against cisplatin-induced AKI

Cisplatin-treated mice demonstrated a steep decline in survival that was mitigated by co-treatment with BARD (Fig. 3). Factors that decreased survival in the Veh/CIS group included loss of kidney function (SCr) and body weight (Fig. S4), and possible dehydration despite an employed saline mitigation strategy (Horn et al. 2013). A similar mouse model administering four weekly doses of 10 mg/kg cisplatin resulted in death within four weeks, but the cause of death was not specified (Katagiri et al. 2016). This coincides with the 88% death rate in the current study at day 29 in mice treated with Veh/CIS.

Kidney injury was assessed by evaluating histopathology, kidney weights, and urinary KIM-1. Tubular injury as assessed by histological changes (cell necrosis, loss of brush border, cast formation, tubule dilatation) was evident in all Veh/CIS mice after just two doses of cisplatin, while 30% of BARD/CIS mice did not display tubular injury or elevated urinary KIM-1 even after four doses of cisplatin suggesting that BARD mitigated cisplatin-induced tubular damage in about one-third of mice (Fig. S1A). The observation of tubular injury after just two doses of cisplatin is consistent with published mouse models of cisplatin-induced kidney injury (Ravichandran et al. 2016). The Veh/CIS mice also had higher levels of apoptosis in the kidney tissue compared to the other groups (Fig. S1B). These results were expected as cisplatin’s mechanism of action of crosslinking DNA strands leads to apoptosis (Oh et al. 2014). The apoptosis was somewhat mitigated, however, by co-treatment with BARD (34% reduction; Fig. S1B). Kidney weights after four doses of cisplatin were 23% less in cisplatin-treated mice compared to vehicle-treated mice (*p* < 0.0001; Fig. S2). Co-treatment with BARD did not significantly alter kidney weights in cisplatin-treated mice (*p* = 0.50; Fig. S2).

Urinary KIM-1 is a specific biomarker of renal proximal tubule injury, increases early following an insult, and has repeatedly outperformed SCr at predicting histopathologic kidney injury, for both low- and high-grade injuries (Vaidya et al. 2010). In cisplatin-treated cancer patients, urinary KIM-1 levels are significantly increased at 3 and 10 days after cisplatin treatment, despite the absence of changes in SCr (George et al. 2017). The results from this mouse model study suggest that increased urinary KIM-1 levels, indicating kidney injury, are apparent with cisplatin treatment even after just one dose, where increases of 299-fold from baseline were observed (Fig. 4A). Opposite of what is typically reported in the literature, the current study found a larger increase in urinary KIM-1 after one dose of cisplatin than after multiple doses. This is likely due to the premature death of some of the cisplatin-treated mice, as the mice with the highest urinary KIM-1 levels after one dose were no longer alive after 3–4 doses of cisplatin. Co-treatment with BARD protected mice from this injury as demonstrated by the 64-fold and 72-fold higher urinary KIM-1 levels observed in Veh/CIS mice after 1 and 3–4 doses of cisplatin respectively, compared to BARD/CIS mice (Fig. 4A). The histopathologic and urinary KIM-1 results confirm that BARD co-treatment mitigates cisplatin-induced kidney injury both early in treatment and after multiple exposures.

Due to convenience and a current lack of FDA approved urinary biomarkers of kidney injury, kidney function is measured in the clinic using SCr with calculation of an eGFR (Inker et al. 2021). The current study demonstrated that co-treatment of cisplatin with BARD prevented significant increases in SCr as the Veh/CIS group demonstrated a 1.9-fold increase in SCr compared to the BARD/CIS group (Fig. 4B). However, clinically, SCr is typically not a good marker for renal function during acute kidney damage as the finding of elevated SCr can be delayed by several days after an insult, SCr increases are observed only after significant reductions (> 50%) in GFR, and SCr is heavily influenced by muscle mass, age, sex, and disease states (Luft et al. 2021). Also, tubular secretion may contribute a larger excretory component of GFR compared to filtration, further confounding the use of SCr to estimate GFR (Eisner et al. 2010). The current study demonstrated kidney injury (assessed by KIM-1) and function (assessed by SCr) results suggesting that BARD helped to mitigate renal injury and function declines observed with cisplatin treatment in mice with cancer.

### Mechanism of BARD nephroprotection

The hypothesized mechanism of BARD nephroprotection includes Nrf2 activation and NF-κB inhibition. Nrf2 is a transcription factor that helps to protect cells from oxidative damage by activating antioxidant genes (Mapuskar et al. 2023; Mirzaei et al. 2021). BARD treatment resulted in a 36-fold increase in GCLC protein levels in kidney tissues compared to mice not treated with BARD (Fig. 5). The BARD-induced increase was partly mitigated by cisplatin exposure as demonstrated by the 49% decrease in GCLC protein levels in the BARD/CIS group compared to the Veh/CIS group (Fig. 5). This is consistent with BARD’s mechanism of action, and other studies have demonstrated kidney increases in GCLC following Nrf2 activation (Lister et al. 2018; Liu et al. 2022; Tanaka et al. 2008; Vaidya et al. 2010). All treatment groups demonstrated low levels of GCLC protein in tumor tissues (data not shown), suggesting that BARD exhibits a preferential renal increase in GCLC which protects against cisplatin-induced kidney injury (Fig. 5). This is a favorable result as increased GCLC protein in the tumor could result in decreased anti-tumor efficacy from cisplatin treatment. The mechanism behind the renal-specific increase of GCLC warrants further analysis, but may be related to varied exposure to BARD or varied Nrf2 expression in the kidneys compared to the tumor.

A previous study reported that BARD increased the expression of the efflux transporter multidrug and toxin extrusion protein 1 (MATE1) in human proximal tubule cells (Atilano-Roque et al. 2016a). This is relevant in the current study as MATE1 is responsible for cisplatin efflux into the urine, suggesting that BARD may increase MATE1 and lead to decreased renal exposure to cisplatin and less subsequent nephrotoxicity. However, the current study did not assess the kidney content or urinary excretion of cisplatin between the treatment groups to test for the functional impact of this mechanism.

Nrf2 activation was non-significantly increased in the kidneys of BARD-treated mice (Fig. S3A). This is likely due to the fact that Nrf2 has a baseline half-life of ~ 20 min and although longer after inhibition of KEAP1, the fact that kidneys and tumors were not collected until 72 h after the final BARD treatment, limited our ability to detect nuclear Nrf2 accumulation in the current study (Ma et al. 2013). Notably, cisplatin treatment resulted in significantly increased Nrf2 activation in tumors likely due to the ongoing cell death of cancer cells (Fig. S3A). Some studies have reported elevated Nrf2 levels in tumor cells that are resistant to cisplatin (Mirzaei et al. 2021; Barroeta et al. 2023). The multiple cisplatin doses may have led to chemoresistance in the tumors of the cisplatin-treated mice, resulting in the observed increase in nuclear Nrf2.

BARD did not significantly alter NF-κB p50 or p65 activation in the kidneys of cisplatin-treated mice. NF-κB is a family of transcription factors that includes p50, p65/RelA, RelB, c-Rel, and p52 that can hetero- and homodimerize to form different dimers (Christian et al. 2016). NF-κB serves roles in regulating the cell cycle, regulating epithelial homeostasis, and activating the immune system (Christian et al. 2016). NF-κB promotes inflammation during renal disease and studies indicate that NF-κB activation is correlated with the severity of renal disease (Fearn et al. 2017). However, most reports are limited to the p50/p65 heterodimer and the function of other NF-κB complexes in renal disease remains unclear. It has been suggested that the p50 subunit has a dual role in both promoting inflammation as a heterodimer (p50/p65) and repressing inflammation as a homodimer (p50/p50) (Fearn et al. 2017). Treatment with BARD resulted in increased NF-κB p50 activation in the kidneys, but no significant differences in NF-κB p50 activation were observed in the tumors (Fig. S3B). This may suggest that the p50 subunit is in fact suppressing inflammation in the kidneys. Treatment with cisplatin resulted in increased NF-κB p65 activation in the kidneys and tumors, which was not significantly mitigated by co-treatment with BARD (Fig. S3C).

### Anti-tumorigenic effects of BARD

Although BARD has been purported to have some anti-tumor effects (Gao et al 2015; Liby et al. 2010; Shishodia et al. 2006; Konopleva et al. 2005, 2002), the study failed to detect any beneficial effects on tumor responses without the addition of cisplatin. However, cisplatin successfully reduced tumor growth compared to vehicle-treated mice and the addition of BARD did not negatively impact the anti-tumor effects of cisplatin (Fig. 6). This latter finding is important as a preferred mitigation tool should not have properties that negatively impact the efficacy of the primary therapy while preventing the toxicity.

### Study Limitations

While the findings of this study are highly encouraging in supporting the potential of BARD as a nephroprotective strategy for cisplatin-induced AKI, there were some limitations that require transparency. Nuclear extracts used to measure Nrf2 and NF-κB activation were prepared using frozen tissue, which typically results in a lower yield than nuclear fractionation with fresh tissue. The cisplatin doses used (10–15 mg/kg), while lower than the single dose mouse model, are still higher than typical doses used in the clinic (< 5 mg/kg). Also, the mice in the study were relatively young (8–10 weeks at start) versus the median older age observed in patients diagnosed with cancer (65 years) (Sharp et al. 2017), somewhat limiting full translation of outcomes from the mouse model to the clinic. Finally, only male mice were examined in this study as the evaluated model of cisplatin-induced AKI was developed using male mice, limiting the assessment of sex as a biological variable. Further studies are needed to fully investigate the multiple mechanisms behind BARD mitigation of cisplatin-induced kidney injury.

## Conclusions

In summary, bardoxolone methyl (BARD) protected against cisplatin-induced AKI in mice with cancer as observed by the KIM-1 urinary biomarker and histopathology. BARD also mitigated the loss of kidney function caused by cisplatin, as measured by SCr. Additionally, BARD did not alter the anti-tumorigenic effects of cisplatin. The results of this pilot study are significant as they demonstrate that BARD could be a potential nephroprotectant to be acutely administered with cisplatin for reducing the risk of AKI. Further studies are warranted to fully evaluate the spectrum of biomarker and pathway changes, as well as potential benefits and possible risks, of BARD as a co-treatment to mitigate the risk of AKI in cisplatin-treated cancer patients.

## Supplementary Material

Supplementary Material

## Data Availability

The datasets generated during and analyzed during the current study are available from the corresponding author on reasonable request.
