# Supplementary material for: Bardoxolone methyl improves survival and reduces clinical measures of kidney injury in tumor-bearing mice treated with cisplatin
Source: AAPS Open. Author manuscript; Available in PMC 2025 Aug 28. (PMC12387945; doi:10.1186/s41120-025-00107-5)
Supplement: Supplementary Material [file NIHMS2075455-supplement-Supplementary_Material.pdf]

Supplementary Materials:

Supplemental Methods

Histological Examination

Paraformaldehyde (4%)-fixed and paraffin-embedded kidneys were sectioned at 4 µm and stained with periodic acid-Schiff (PAS) by standard methods. All histological examinations were performed by a blinded renal pathologist. Histological changes due to tubular injury were evaluated in the outer stripe of the outer medulla on PAS-stained tissue and were quantified by counting the percent of tubules that displayed cell necrosis, loss of brush border, cast formation, and tubule dilatation as follows: 0 = none, 1 = <25%, 2 = 26-50%, and 3 = >50%. At least 10 high power fields (HPFs; x200) were reviewed for each slide.

4 µm sections of paraffin-embedded kidneys were prepared for immunodetection of cleaved caspase-3 (Cell Signaling, Danvers, MA; polyclonal; 1:500; 100 µL per slide). Antigens were revealed in pH 9.5 BORG solution (Biocare Medical, Concord, CA) for 10 minutes at 110°C (NxGen Decloaker, Biocare Medical, Concord, CA) with a 10 minute ambient cool down. Immunodetection was performed on the Discovery Ultra stainer (Ventana/Roche, Indianapolis, IN) with primary incubation for 32 minutes at 37°C. Caspase-3 was visualized with the OmniMAP anti-rabbit HRP and ChromoMAP DAB kit (Ventana/Roche, Indianapolis, IN; 37°C for 12 minutes for each reagent). All sections were counterstained in Harris hematoxylin for 2 minutes, blued in 1% ammonium hydroxide, dehydrated in graded alcohols, cleared in xylene and cover glass mounted

using synthetic resin. Negative controls to confirm the specificity of the immunostaining included omission of the primary antibody incubation step in the immunohistochemistry (IHC) protocol and substitution of the primary antibody diluent. Cleaved caspase-3 IHC was used to identify apoptotic bodies in the kidney tissue. At least 10 HPFs (x200) were reviewed for each slide and the number of apoptotic bodies was averaged across the 10 HPFs.

Analyses for differences between treatment groups for histopathological tubular injury were assessed by rank-order two-way ANOVA with Tukey-Kramer post-hoc test using GraphPad Prism. Analyses for differences between treatment groups for histological apoptotic body counts were assessed by two-way ANOVA with Tukey-Kramer post-hoc test using GraphPad Prism.

Sample Assays

Nuclear extracts were isolated from mouse kidney and tumor samples collected at the study end using a nuclear extract kit (Cat. #40010, Active Motif, Carlsbad, CA). Activated Nrf2 and activated p50 and p65 NF-κB were measured in the nuclear extracts using DNA-binding ELISA kits (TransAM® Nrf2, Cat. #50296; TransAM® NF-κB Family, Cat. #43296, Active Motif, Carlsbad, CA).

Analyses for differences between treatment groups for activated Nrf2 levels and activated NF-κB levels were assessed by two-way ANOVA with Tukey-Kramer post-hoc test using GraphPad Prism.

Figure Legends:

Figure S1: Bardoxolone methyl (BARD) mitigation of histopathological kidney injury in cisplatin-treated mice. All mice received 4 doses unless requiring euthanization due to humane endpoints. Mice sacrificed after 3 and 4 doses of cisplatin were combined to increase available sample sizes. (A) Tubular injury score of kidneys from mice following 3-4 doses of cisplatin for each treatment group (n=2-9/group). Scoring criteria: 0 = none, 1 = <25%, 2 = 26-50%, and 3 = >50. Data are represented as median; *p≤0.05. (B) Average apoptotic body count per high power field (HPF) of kidneys from mice following 3-4 doses of cisplatin for each treatment group (n=2-9/group). Kidney samples were IHC stained for cleaved caspase-3 and apoptotic bodies were counted in 10 HPFs per sample. Data represented as mean ± standard deviation (SD). (C) Representative PAS-stained kidneys from each treatment group after four doses of cisplatin (200x magnification). (D) Representative cleaved caspase-3 IHC-stained kidneys from each treatment group after four doses of cisplatin (200x magnification). Apoptotic bodies (*arrows*) are stained brown. (E) Some cisplatin-treated mice displayed PAS-positive intranuclear inclusions (*arrows*) in tubular cells (400x magnification).

Figure S2: Cisplatin treatment resulted in smaller kidneys. (A) Average kidney weights for each treatment group (n=16-20 kidneys/group). Mice treated with cisplatin had significantly smaller kidneys as compared to non-cisplatin-treated mice (p<0.0001). Veh/CIS and BARD/CIS mice had similar kidney weights (p=0.50). (B) Average kidney weights for each treatment group normalized by body weight at sacrifice (n=16-20 kidneys/group). Kidney weights were measured at sacrifice after four doses of cisplatin.

Data represented as mean ± standard deviation (SD); **p≤0.01, ***p≤0.001, ****p≤0.0001.

Figure S3: Nrf2 and NF-κB activation in cisplatin and BARD-treated mice. Relative Nrf2 and NF-κB activation in kidney and tumor tissues for each treatment group (n=4-5/group). (A) Nrf2. (B) NF-κB p50. (C) NF-κB p65. Data represented as mean ± standard deviation (SD); *p≤0.05, **p≤0.01.

Figure S4: Cisplatin treatment resulted in significant loss of body weight, even after just one dose. Data represented as mean ± standard deviation (SD); *p≤0.05, **p≤0.01, ***p≤0.001, ****p≤0.0001.

Table Headers:

Table S1: Baseline values by treatment group.

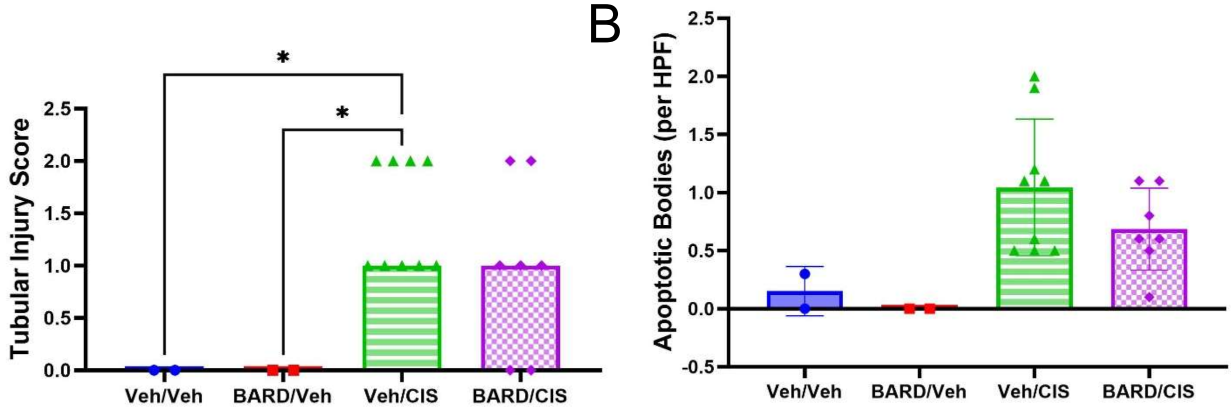

C

	**BARD vehicle**	**BARD**
**Cisplatin vehicle**	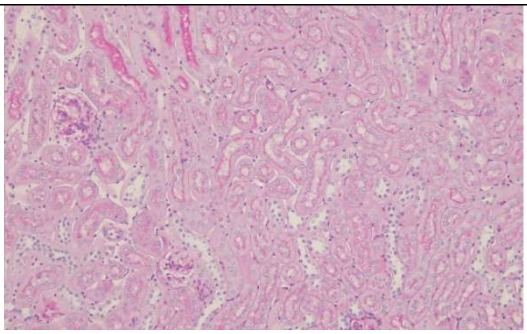 Tubular injury score: 0	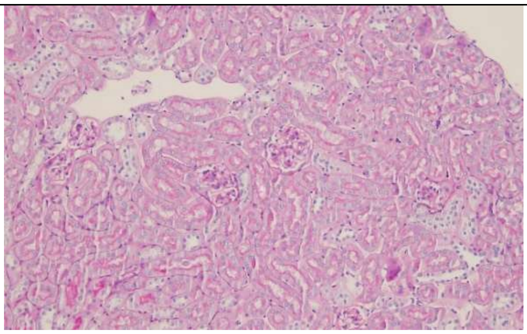 Tubular injury score: 0
**Cisplatin**	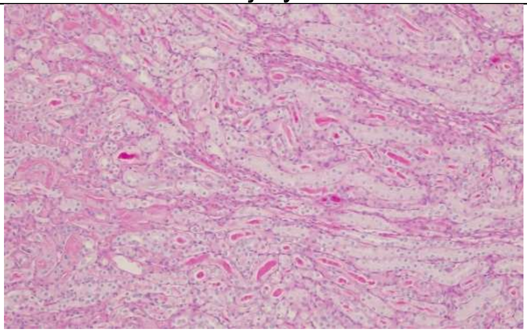 Tubular injury score: 2	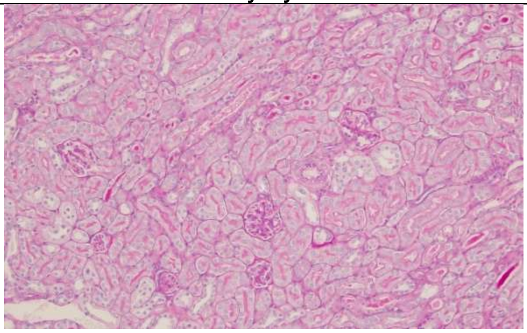 Tubular injury score: 1

D

	BARD vehicle	BARD
**Cisplatin vehicle**	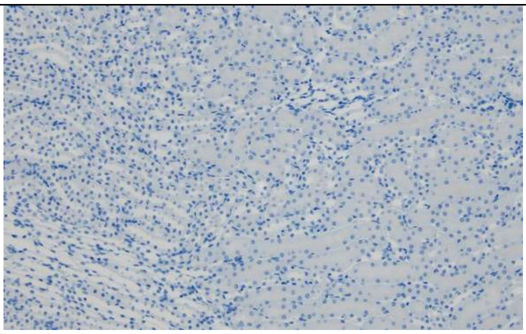 Average apoptotic bodies per HPF: 0	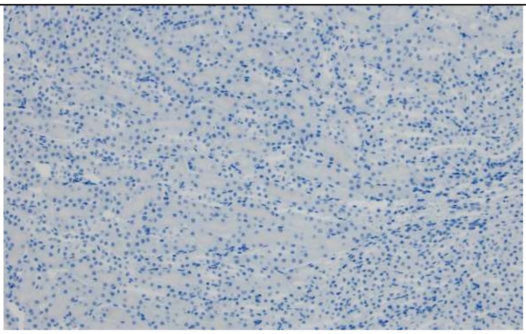 Average apoptotic bodies per HPF: 0
**Cisplatin**	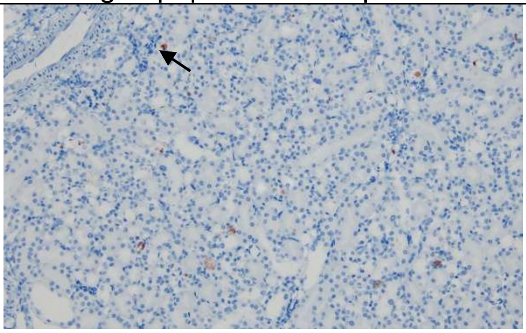 Average apoptotic bodies per HPF: 2.0	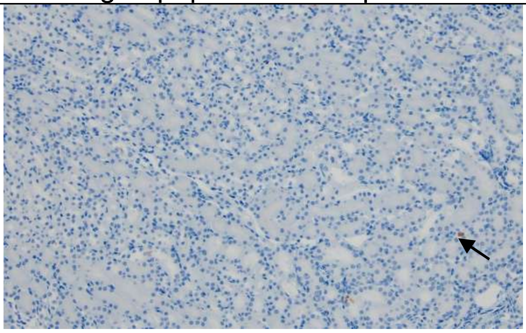 Average apoptotic bodies per HPF: 0.5

E

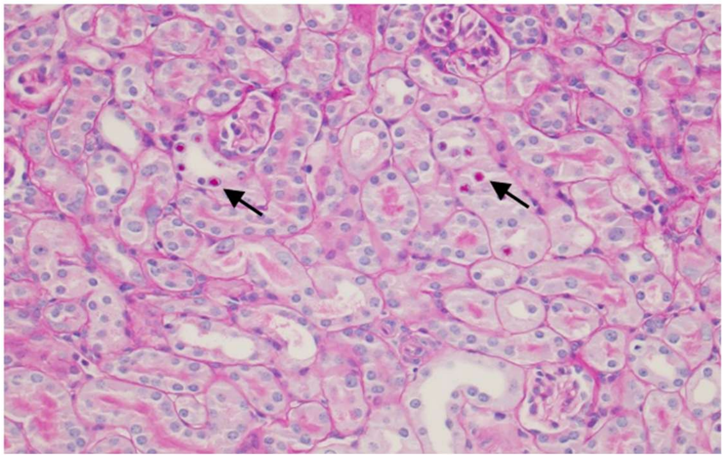

Figure S1

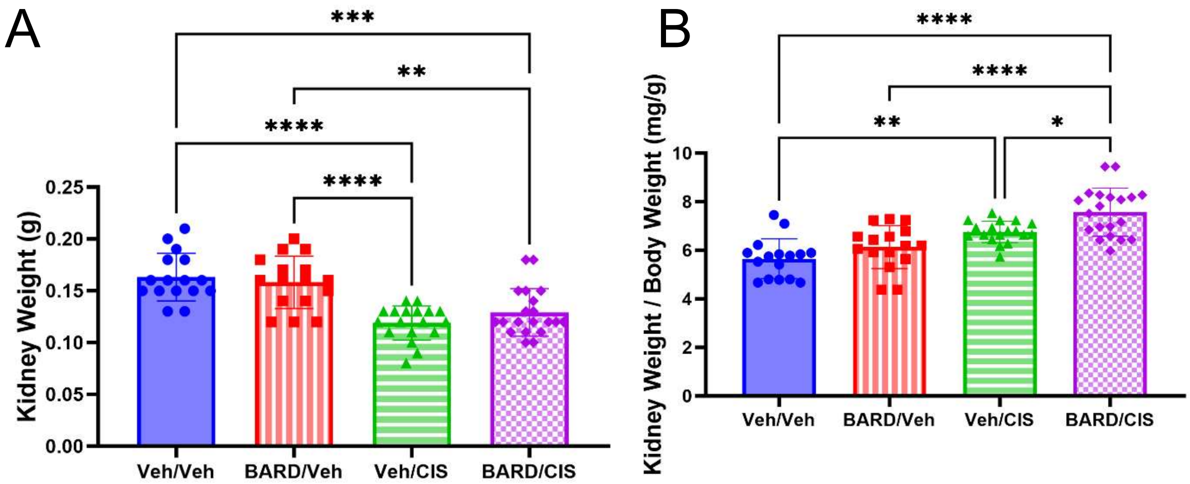
A
***
**
****
****
Kidney Weight (g)
0.25
0.20
0.15
0.10
0.05
0.00
Veh/Veh
BARD/Veh
Veh/CIS
BARD/CIS
B
****
****
**
*
Kidney Weight / Body Weight (mg/g)
10
8
6
4
2
0
Veh/Veh
BARD/Veh
Veh/CIS
BARD/CIS

Figure S2

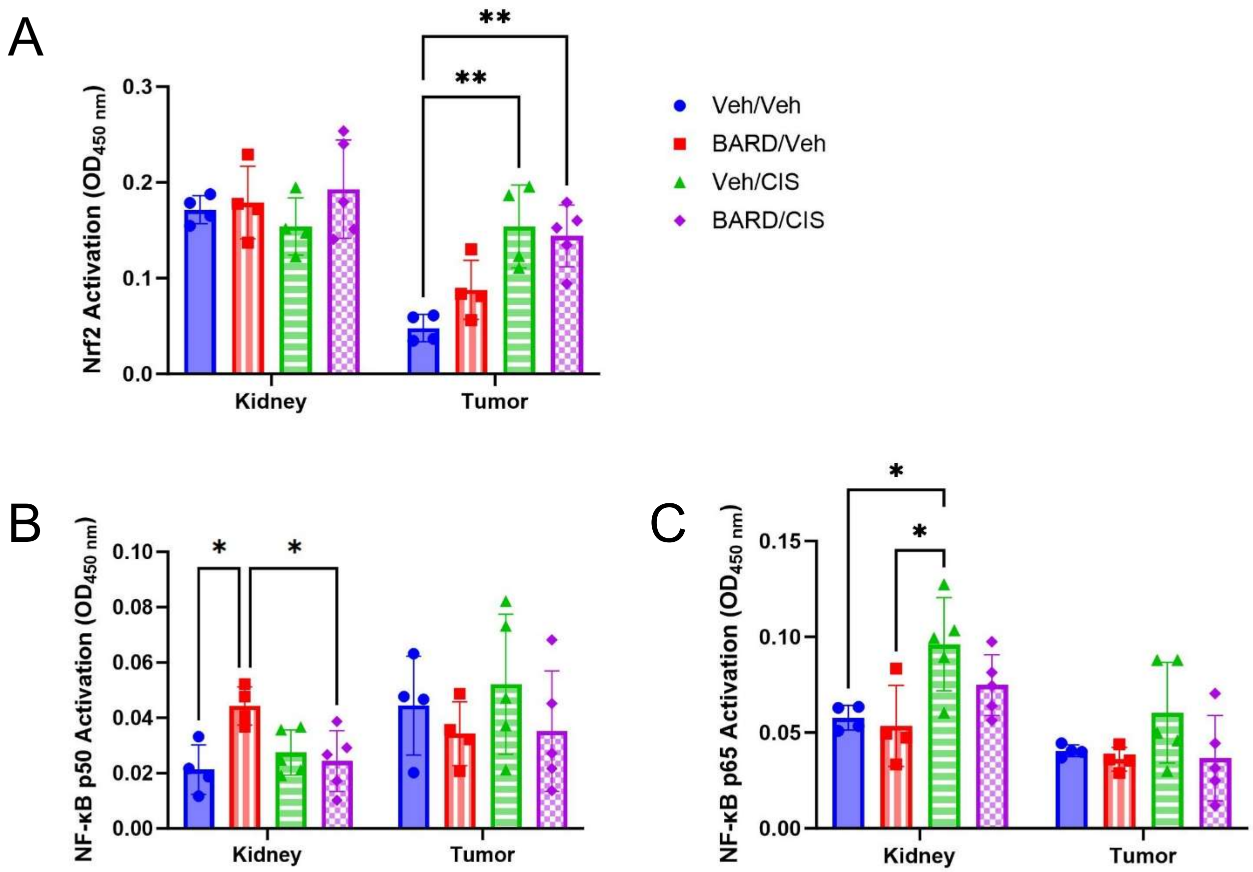
A
**
**
Nrf2 Activation (OD450 nm)
0.3
0.2
0.1
0.0
Kidney
Tumor
Veh/Veh
BARD/Veh
Veh/CIS
BARD/CIS
B
*
*
NF-κB p50 Activation (OD450 nm)
0.10
0.08
0.06
0.04
0.02
0.00
Kidney
Tumor
C
*
*
NF-κB p65 Activation (OD450 nm)
0.15
0.10
0.05
0.00
Kidney
Tumor

Figure S3

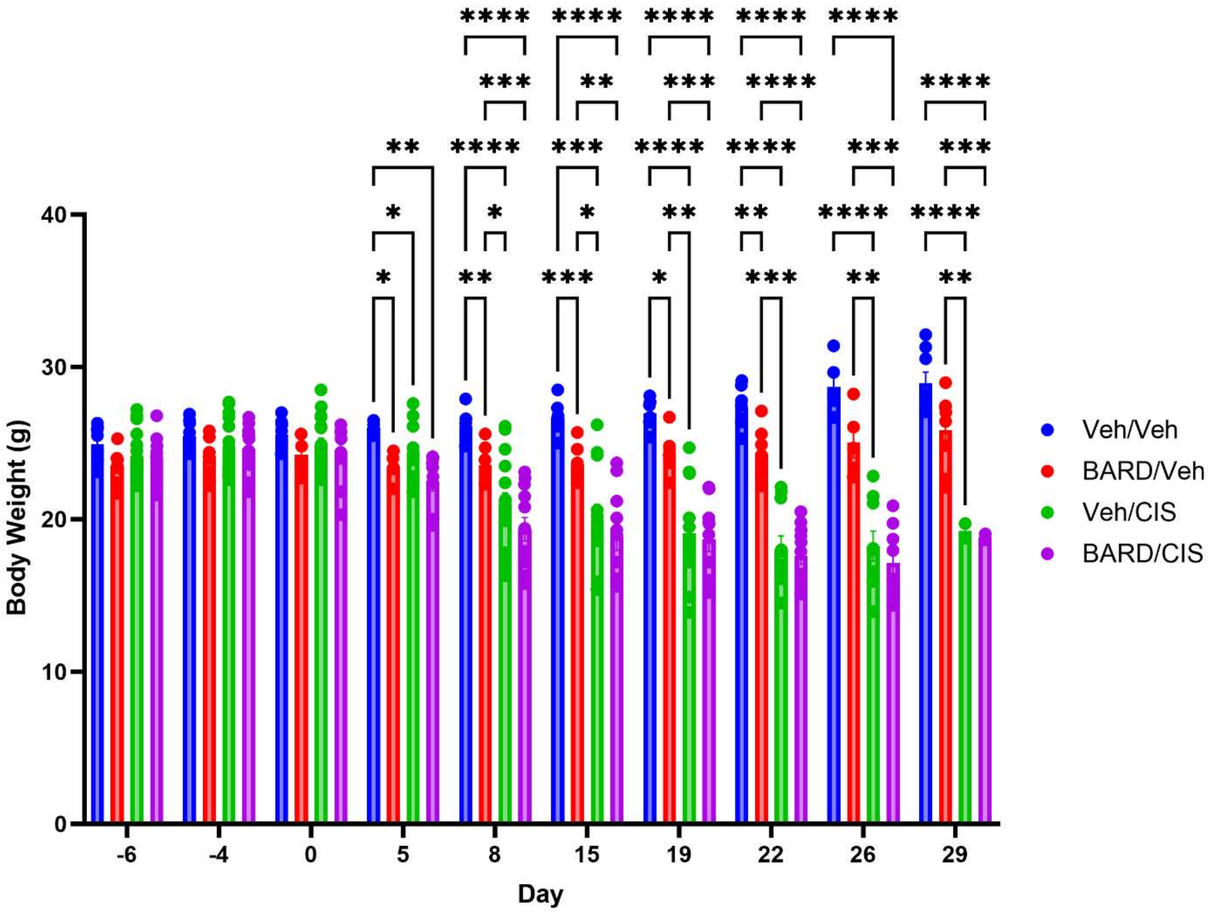

Body Weight (g)
40
30
20
10
0
-6
-4
0
5
8
15
19
22
26
29
Day
Veh/Veh
BARD/Veh
Veh/CIS
BARD/CIS

Figure S4

Table S1

Group	Veh/Veh (n=8)	BARD/Veh (n=8)	Veh/CIS (n=16)	BARD/CIS (n=12)
**Baseline body weight (g)**	24.9 ± 1.2	23.5 ± 1.4	24.2 ± 1.6	24.1 ± 1.5
**Baseline tumor volume (mm^3)**	0.88 ± 1.36	0.25 ± 0.46	0.25 ± 0.77	0.58 ± 1.17

Data represented as mean ± standard deviation (SD). Veh: vehicle; BARD: bardoxolone methyl; CIS: cisplatin.
